# A healing justice approach to grief in communities of color

**DOI:** 10.3389/fpsyt.2025.1508177

**Published:** 2025-04-10

**Authors:** Michelle Chang

**Affiliations:** Department of Psychology, University of California, Los Angeles, Los Angeles, CA, United States

**Keywords:** grief, loss, healing justice, people of color (POC), grief intervention, collective grief, ritual, protest

## Introduction

Following the deaths of over 1 million people from COVID and record-breaking deaths per year from police brutality in the U.S., the toll of grief from unexpected, sudden, and traumatic deaths is immense in communities of color. They have moreover been long overburdened with losses of loved ones due to structural racism, including lower income earnings (1), incarceration (2), exposure to pollutants (3), residential segregation (4), food insecurity (5), and higher eviction rates (6) that contribute to earlier death among people of color. A programmatic response to address the psychological impact of loss is imperatively necessary. Researchers, clinicians, and public health officials have called for clinical resources and interventions to support bereaved individuals, given high comorbidities of complicated grief, PTSD, and depression among those mourning losses (7, 8). However, clinical frameworks of symptom reduction may be insufficient in meeting what is required of grief. To chart a path forward alongside grief necessitates an approach grounded in healing strategies defined by the collective transformation of conditions of death-making and rooted in long-held ancestral wisdoms of resistance.

## Current grief treatments

The current gold standard treatments for grief are evidence-based psychotherapies, delivered in individualized and group settings. For example, cognitive-behavioral therapy (CBT) provides psychoeducation around normal and pathological grief, addresses rumination and avoidance, identifies and expresses emotions related to loss, and implements coping strategies (9). Interpersonal therapy (IPT) addresses grief by focusing on a specific interpersonal problem, thus increasing bereaved individuals’ capacity to connect in relationships and accept social support (10). Complicated grief treatment (CGT), on the other hand, combines elements of CBT and IPT, such as prolonged exposure, in service of psychoeducation, goal-setting, and narrativization of grief (11). And still, some support groups generally facilitate adjustments following the loss of a loved one across emotional and practical skills or provide unstructured, designated space for processing in community (12). These services provide relief for bereaved individuals, such as by improving abilities to engage in meaningful relationships and activities even after loss and by normalizing and validating the emotional experience of grief (13). Clinicians and mental health providers recognize that providing the tools for symptom reduction of grief, trauma, depression, and other mental health challenges following bereavement is necessarily a core component of our work.

However, these therapies have predominantly been researched in U.S. and European contexts (14). Meta-analyses of grief-focused interventions consistently find that participant race or ethnicity is altogether not reported in many existing studies (15, 16), suggesting that treatment approaches for grief have not been developed or researched while centering the lived experiences of communities of color. We must importantly consider the implications of mental healthcare responses to grief that solely measure the success of a treatment through the alleviation of psychological symptoms such as intense feelings of anger and emotional pain or difficulties reintegrating with activities. When used to shield society from the discomfort of seeing people in pain from loss or to normalize loss decontextualized from its unjust and structurally racist antecedents, symptom reduction may simultaneously serve as a stabilizing and pacifying force in maintaining the conditions that create premature death and disproportionate grief among racially oppressed people.

## Healing justice: grief as resistance

What can we in fact learn from the bereaved who are so-called “treatment-resistant,” who have “chronic,” unremitting trajectories of grief, or who still struggle to “reintegrate” with the world following loss? Outside of traditional psychological studies, community organizers provide many examples of years-long grief that persists; those bereaved from unjust and premature death have often been at the forefront of movements and protests such as Black Lives Matter (17). There is much to learn from the loved ones of 23-year-old Black woman Shantel Davis; they brought a coffin bearing her name to city hall so that local officials could witness their grief after she was slain by police (18). There is much to learn from the villagers and neighbors of Salim, a Palestinian father whose infant was asphyxiated to death by tear gas; together, they refused for four hours to concede to the military blockade of the graveyard meant to be his infant’s burial grounds (19, 20). There is much to learn from the family members who have led protests and vigils for years outside Men’s Central Jail in Los Angeles, where their predominantly Black and Latinx loved ones unjustly died in negligence while awaiting trial (21, 22). There is much to learn from the Skid Row community members who constructed public memorials at the site of the murder of Black Skid Row resident Charly “Africa” Leundeu Keunang by Los Angeles police (23). These bereaved individuals encourage us to attend to, sit with, and continuously return to the pain of loss.

By recognizing grief as intertwined with resistance, these examples reveal a different framework of focus for responses to loss through *healing justice*, which is a “community-led response to intervene and interrupt individual and generational trauma while building collective power towards resistance” to sustain our “emotional, physical, mental, spiritual, psychic, & environmental wellbeing” (24). Under Black abolitionists’ conceptualization (24), healing justice has long provided communities of color with the blueprints for understanding grief as a politicized praxis of care, demanding that the lives of the deceased must fundamentally be seen as precious, important, and indispensable. Bereaved loved ones set visions for a more livable world in which their loved ones could have instead lived long, full lives (25). Care in grief thus necessarily translates into care for the present and future generations of the living.

Community rituals present another such realization of healing justice applied to grief. They serve critical functions to deindividualize grief, incorporate the bereaved into the social fabric, reaffirm cultural beliefs, and publicly sanction emotional pain (26). These rituals may draw from ancestral wisdoms and technologies that preceded Eurocentric conceptualizations of evidence-based grief treatments. Bereaved community members often incorporate their own culturally relevant rituals into social movements (27), such as singing ancestral songs from their lineages or building communal altars at protests. And still, sites of political mobilization may even facilitate the formation of new rituals altogether.

Yet healing justice centered responses to grief may likely be the first to be dismissed by academics, care providers, and state actors such as police. Under a racial capitalist state, rituals—and the time required to tend to them—have become increasingly inaccessible due to their resulting losses in productivity and economic growth (28). The social movements that the bereaved lead are similarly rendered disruptive (29), potentially threatening status quos of oppression and involving tactics (e.g., walk-outs, blocks to street traffic) that materially interrupt work and productivity. Especially when enacted by bereaved people of color, healing justice approaches to grief are subject to repression.

## Discussion and recommendations

Psychologists, mental health providers, and researchers have a significant role to play in legitimizing rituals, protest, and other knowledges as forms of healing alongside grief beyond psychotherapy interventions. Within clinical practice for mental health providers, a healing justice framework integrates a sociopolitical analysis of the conditions preceding loss in mediums across clinical documentation and case conceptualization of clients’ presenting concerns. Rather than solely pathologizing symptoms of grief, providers can support existing ways that bereaved people of color may already be engaging their grief with healing justice. Providers may also produce letters of support or accommodation for bereaved individuals of color to access paid leave and adjustments in their work in order to enable participation in rituals or political actions.

Beyond individual care, the fields of psychology and public health must advocate for workplace and school settings to institutionalize and standardize paid bereavement leave policies or crisis funds for bereavement support that facilitate access to rituals and political actions. For example, of over 3500 higher education institutions in the U.S., less than 1% have bereavement policies for students (30). Psychologists, practitioners, and public health workers moreover hold a responsibility for our work to contribute to dismantling the structural violence that propels racially oppressed communities to sudden and premature death. We must align ourselves with political movements by building power with existing coalitions and organizations that facilitate community action toward more livable worlds embedded in healing justice. Such community action ranges from abolishing surveillance programs that incentivize counselors to criminalize and report youth in Los Angeles schools to armed school police forces (31), to community models of defense against violent sweeps of unhoused residents. In doing so, we can support the autonomy of community-led responses to grief that render obsolete hyperindividualized, de-politicized treatment approaches.
